# Metagenome-assembled genome of *Zalaria obscura* strain JY119

**DOI:** 10.1128/mra.00786-23

**Published:** 2024-04-30

**Authors:** Lakshmanan Vighnesh, Uppada Jagadeeshwari, Chintalapati Sasikala, Chintalapati Venkata Ramana

**Affiliations:** 1 Department of Plant Sciences, School of Life Sciences, University of Hyderabad, Hyderabad, India; 2 Smart Microbiological Services, Hyderabad, Telangana, India; University of California Riverside, Riverside, California, USA

**Keywords:** *Zalaria obscura*

## Abstract

Here, we report a 22.1-Mbp genome sequence of microcolonial fungi, *Zalaria obscura*, isolated from a pine tree bark. The microbiome of the new fungi is predicted to be largely associated with *Acidobacteriota*. The genome sequence of *Zalaria obscura* will help us in understanding the unusual relationship with *Acidobacteriota* member(s).

## ANNOUNCEMENT

*Zalaria obscura* strain JY119, a member belonging to the class *Dothideomycetes* of the phylum *Ascomycota*, a microcolonial fungus, was isolated from *Pinus roxburghii* bark from Meghalaya, India (25.3443°N 91.5335°E). The sample was enriched in Yeast extract Peptone Dextrose (YPD) broth for 3–5 days, and further colonies were isolated and purified on YPD agar plates followed by identification based on D1/D2 sequencing (1). Amplicon sequencing showed that *Acidobacteriota* were the dominant phylum of the microbiome associated with strain JY119 (65%) whereas *Bacillota* dominated in other yeast strains analyzed in the same study (1). The presence of acidobacteria in class *Dothideomycetes* is interesting because of their unusual niche which was previously undiscovered (2, 3). For having a better understanding of the above, we further performed shotgun-based metagenome sequencing of the strain JY119.

A single colony was inoculated into YPD medium containing yeast extract (1%), peptone (2%), and dextrose (2%) and incubated under shaking conditions at 28°C and sent for sequencing to Eurofins Genomics India Pvt. Ltd. (India). Metagenomic DNA was isolated using the Quick-DNA Miniprep Plus Kit; quality check and quantification were performed using NanoDrop. The paired-end sequencing libraries were prepared using the Illumina TruSeq Nano DNA Library Prep Kit, and libraries were filtered using on Agilent 4200 Tape Station. The filtered paired-end libraries were then loaded onto NextSeq500 for cluster generation and sequencing using 2 × 150 bp chemistry. The sequencing generated 7,224,014,295 bp (7.22 Gb). The raw data were then processed using tools and apps available in the KBase platform (4). First, the read quality was checked using FastQC (v0.11.9; https://www.bioinformatics.babraham.ac.uk/projects/fastqc/), followed by trimming the reads using Trimmomatic (v0.36) (5). After trimming, 7.13 Gb which was of good quality was used for further analysis. The reads were then assembled using metaSPADES (v3.15.3) (6). Binning was performed using MaxBin2 (v2.2.4) (7). The obtained metagenome-assembled genome (MAG) was deposited in NCBI database. BUSCO (v5.4.6) (8) which was available in the Galaxy web platform (9) was used to assess the genome assembly quality and completeness using the dothideomycetes_odb10 data set. Additionally, the metagenome reads after trimming were classified using Kaiju (v1.7.3.) (10) to assess the bacterial diversity. A phylogenomic tree was constructed using the available representative genomes (in NCBI) of the order *Dothideales* (11) using the UFCG pipeline (v1.0.2) (12). All the tools were ran using the default parameters, and their versions are indicated in parentheses.

**Fig 1:**
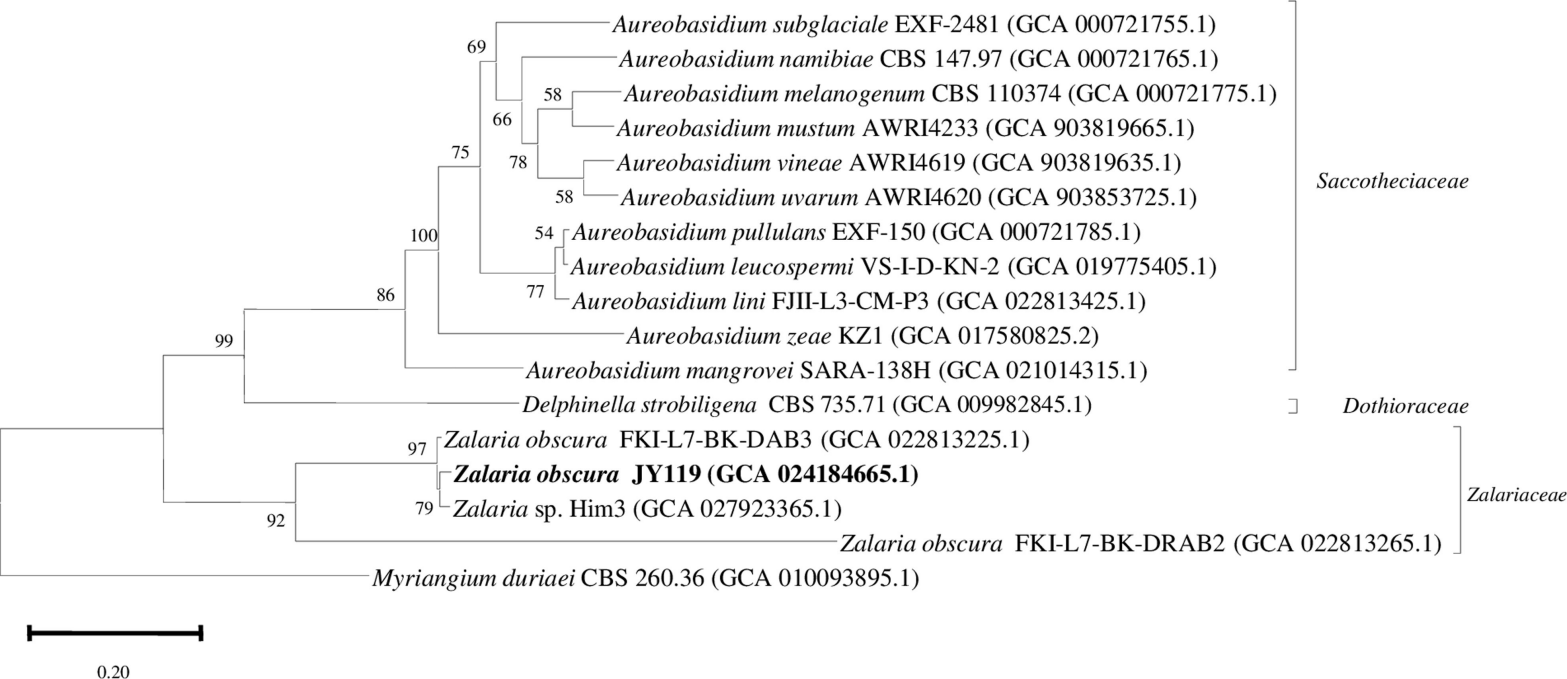
Maximum likelihood-based phylogenomic tree of members closely associated with the genus *Zalaria*. Genome sequence of *Myriangium duriaei* CBS 260.36 is taken as outgroup. The GenBank accession numbers for genome sequences are shown in parentheses. Bar,0.20 nucleotide substitution per position.

The MAG obtained from strain JY119 had a genome size of 22.1 Mb with 53.5% GC content and is phylogenomically related to *Zalaria obscura* (Fig. 1). The number of contigs is 67 and contig N50 is 579,019 bp. Out of 3,786 BUSCOs, 96.5% was complete and single-copy BUSCOs. The kaiju classification predicted about 1% of the reads belongs to the phylum *Acidobacteriota*, which aids in examining the connection with *Zalaria obscura*.

## Data Availability

The MAG has been deposited in GenBank under the accession number JAMKPW000000000, with BioProject and SRA accession numbers PRJNA833239 and SRX15207383, respectively.
